# Choledochal Cyst in Pregnancy: A Case Report

**DOI:** 10.7759/cureus.29774

**Published:** 2022-09-30

**Authors:** Ahmed I Elmasry, Shaher M Ali, Dana A Neama, Alaa M Marzooq

**Affiliations:** 1 General Surgery, Salmaniya Medical Complex, Manama, BHR

**Keywords:** gall bladder disease, pregnancy, cholecystitis, type iv a, choledochal cyst

## Abstract

Choledochal cyst is a rare congenital malformation of the biliary tree. It can be present in various locations along the biliary tree. The diagnosis of choledochal cyst during pregnancy can be challenging for clinicians due to its atypical presentation. In this case report, we discuss a case of a female patient who presented in the third trimester. She was misdiagnosed with cholecystitis and was treated medically. She underwent emergency lower segment cesarean section (LSCS) and was then discharged. The patient later presented with the same symptoms and was diagnosed with choledochal cyst type IVA. She underwent percutaneous drainage and improved. The patient had to deliver the baby prematurely due to the late diagnosis and thus late treatment.

In order to avoid the recurrence of those events, physicians should be familiar with the presentation of a choledochal cyst and should subsequently use the proper imaging modalities such as MRI more frequently in pregnant patients with such presentation, which will result in an early diagnosis and prevent the maternal and fetal complications.

## Introduction

Choledochal cyst is a rare congenital malformation of the biliary tree [1]. It can appear in various locations along the biliary tree depending on the type, which was first described in 1959 by Alonso-lej et al., who classified it into three types of duct dilation [2]. Todani et al. modified the classification in 1977 into four types [3], which are most commonly used by clinicians nowadays. The diagnosis of a choledochal cyst during pregnancy can be challenging for clinicians due to its atypical presentation, limitations of radiographic modalities, and hormonal effects on the biliary system [4]. In this article, we present a case of a female patient with a choledochal cyst that first presented during pregnancy and was misdiagnosed before being diagnosed post-partum.

## Case presentation

First admission

A 32-year-old Filipino female patient presented with no history of medical illness. She is a primigravida at 30+0 weeks. She was referred to our tertiary care hospital from a private clinic presenting with acute non-radiating abdominal pain, mostly located in the right upper quadrant and epigastric area. She reported symptoms of nausea and vomiting twice but denied having any history of fever, diarrhea, or changes in her urine or stool. On physical examination, she was in severe pain but vitally stable. On abdominal examination, there was generalized abdominal tenderness. However, it was difficult to further assess the abdomen due to the gravid uterus. Bloodwork included complete blood count, liver function tests, and amylase enzyme (Table 1).

**Table 1:** First admission laboratory results

Parameters	First Admission Laboratory Values	Reference Range of Salmaniya Medical Complex Laboratory
			White blood count (10^9^/L)	10.26	3.6–9.6
Hemoglobin (g/dL)	11	12.0–5.2
Platelets (10^9^/L)	249	150.0–400.0
Bilirubin-total (µmol/L)	39	5–21
Bilirubin-direct (µmol/L)	29	0–5
Bilirubin-indirect (µmol/L)	10	<18
Alanine transaminase (U/l)	180	<33
Alkaline phosphatase (U/l)	136	50–136
Gamma-glutamyl transferase (U/l)	66	5–55
Amylase (U/L)	147	30–118
Prothrombin time (sec)	12.8	10–14
International normalized ratio (INR)	1.07	0.61–1.17

**Figure 1:**
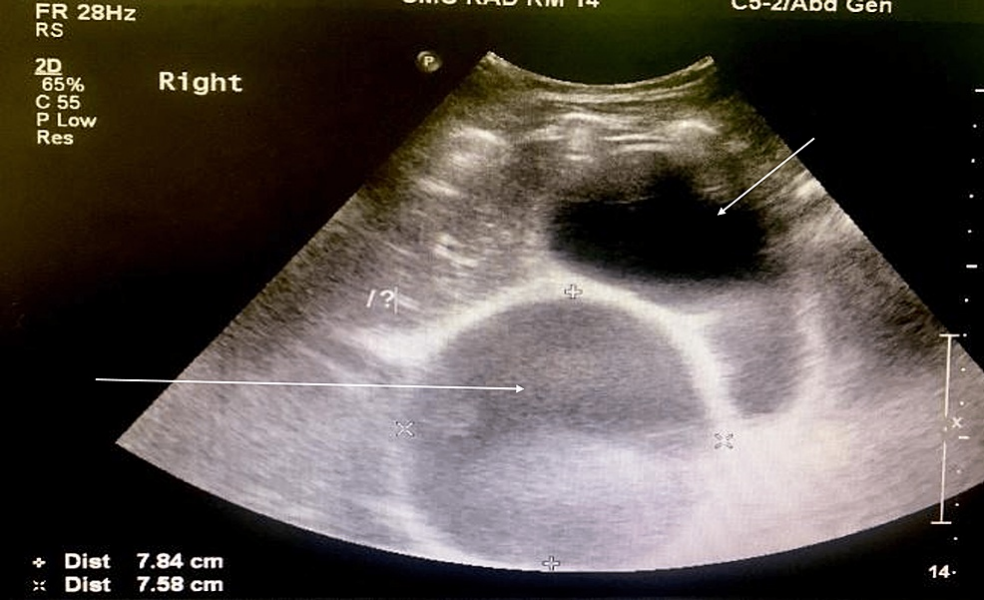
Abdominal ultrasound The short white arrow shows the distended gallbladder, and the long white arrow shows the cyst measuring about 7.8 x 7.6 cm.

Abdominal ultrasound was done, but it was inconclusive due to the distended gravid uterus compressing most of the abdominal organs. It showed a gallbladder significantly distended, with a thickened wall of 0.42 cm containing sludge and tiny stones. There was no obvious intrahepatic biliary dilation; however, there was a large cystic structure measuring 7.8 x 7.6 cm in close proximity to the gallbladder. It was unclear if the cyst was ballooning the gallbladder or if it was a common hepatic duct cyst (Figure 1). The patient was admitted under the care of obstetrics and gynecology for observation but showed no improvement in symptoms. She was started on an antibiotic. The patient underwent emergency lower segment cesarean section (LSCS) due to preterm labor and fetal distress, with no intra- or postoperative complications. Despite a slightly abnormal liver function test (LFT), the patient was discharged to be followed up.

Second admission

The same patient was referred again to our hospital from a private clinic one day after discharge. In the private clinic, she had an abdominal ultrasound that revealed a choledochal cyst. Upon history taking, she mentioned that her symptoms had deteriorated. She complained of severe abdominal pain in the epigastric region, with a severity score of 9/10 as well as nausea and vomiting. She denied having any other symptoms such as fever, pruritus, or changes in her urine or stool. She had mild jaundice with abdominal distension on physical examination, and no mass was felt upon palpation. There was severe tenderness in the right hypochondriac and epigastric regions, with negative Murphy’s sign, and no other findings were noted on systemic examination. Bloodwork included complete blood count, liver function tests, amylase enzyme, and Hepatitis profile (Table 2).

**Table 2:** Second admission laboratory results

Parameters	Second Admission			Laboratory Values at Last Follow-Up	Reference Range of Salmaniya Medical Complex Laboratory
	Pre-drain Insertion Laboratory Values	Post-drain Insertion Laboratory Values	Post-stent Insertion Laboratory Values		
White blood count (10^9^/L)	8.85	6.24	6.5	6.4	3.6–9.6
Hemoglobin (g/dL)	8.3	11.2	11.1	11	12.0–5.2
Platelets (10^9^/L)	548	548	425	302	150.0–400.0
Bilirubin-total (µmol/L)	98	82	37	24	5–21
Bilirubin-direct (µmol/L)	79	64	31	7	0–5
Bilirubin-indirect (µmol/L)	19	18	6	17	<18
Alanine transaminase (U/l)	125	55	30	28	<33
Alkaline phosphatase (U/l)	930	691	212	135	50–136
Gamma-glutamyl transferase (U/l)	1193	971	195	60	5–55
Amylase (U/L)	250				30–118
Prothrombin time (sec)	21	19.7	20		10–14
International normalized ratio (INR)	1.79	1.64	1.7		0.61–1.17
Hepatitis profile	Negative				

**Figure 2:**
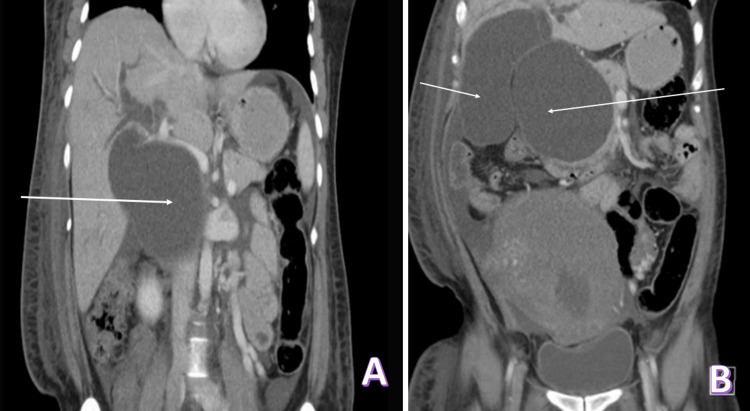
(A) Coronal view: The long arrow white shows a choledochal cyst (type IVA). (B) Sagittal view: The long white arrow shows a choledochal cyst (type IVA), and the short arrow shows CBD 12 x 8.7 x 10 cm (RL x AP x CC). CBD: Common bile duct.

Abdominal computed tomography (CT) showed multiple cystic intrahepatic and large extrahepatic ducts that were dilated and communicating with the biliary tree. Furthermore, the intrahepatic ducts were measuring about 1.4 cm, and the common bile duct (CBD) was about 12 x 8.7 x 10 cm (RL x AP x CC). The findings were suggestive of a choledochal cyst (type IVA). The gallbladder and cystic duct were dilated with no stones noted. The pancreas was normal in size with no signs of acute pancreatitis (Figure 2). The patient underwent percutaneous external biliary drain insertion under ultrasound guidance; the drain was inserted through the right liver biliary system, with its tip lying within the large hilar cyst. The patient started to improve clinically with the drain yielding about 1300-1800 ml of bile daily.

After two weeks, she underwent internal stent insertion through endoscopic cholangiopancreatography guidance via the ampulla of Vater with the removal of external drainage. As she showed improvement clinically and in bloodwork tests (Table 1), she was discharged with follow-up. The patient was reviewed two weeks after discharge and reported that her symptoms had improved. Her liver function tests showed a reduction in liver enzymes as well as resolution of jaundice and abdominal pain. Repeated abdominal US was requested, and her report showed regression of the choledochal cyst's size compared to the previous abdominal US, measuring about 6 x 1.8 cm with a stent seen within. However, the patient decided to go back to her home country to continue her treatment.

## Discussion

Choledochal cyst is a rare congenital anomaly of the bile tree with a prevalence of one in 100,000-150,000 in the United States of America (USA) [5]. Almost 75% of choledochal cyst cases are diagnosed during childhood, with the remaining 25% of the cases being diagnosed later in life [6]. Females account for the majority of cases, with a ratio of 4:1 and 3:1 for females and males, respectively [6].

That being said, the diagnosis of a choledochal cyst during pregnancy poses a challenge due to the rare presentation in adulthood [6] and gallbladder function changes caused by pregnancy. Such changes include residual and fasting volumes in the gallbladder, which are twice higher in pregnant females when compared to non-pregnant females of the same age. Furthermore, the gallbladder empties in a much slower manner than in non-pregnant controls, which mimics the effects of a choledochal cyst on the gallbladder [7].

One of the main challenges that surgeons face when diagnosing a choledochal cyst in pregnant patients is the atypical and vague presentation [8]. The diagnosis in children may be easier due to the classic triad of choledochal cyst, which is abdominal pain, jaundice, and right upper quadrant abdominal mass [9]; however, symptoms are much more vague in adult patients, let alone pregnant patients [8]. Most patients present with abdominal pain as the chief complaint, while only one-third of patients present with the classic triad [9].

Another difficulty that physicians face during the diagnosis of choledochal cysts in pregnant patients is the limited use of radiological modalities. While ultrasonography is a perfectly safe option during pregnancy, it is not very useful due to the changes in the abdominal anatomy that happen during pregnancy and the gravid uterus effects. Additionally, the anatomical details of the biliary tree cannot be assessed by ultrasound [10]. Useful imaging modalities such as CT and endoscopic retrograde cholangiopancreatography (ERCP) are of limited use during pregnancy due to the risk of exposing the fetus and the mother to ionizing radiation [11]. In contrast, non-radioactive imaging techniques, such as magnetic resonance imaging (MRI), are safe to use during pregnancy, and it provides a clear anatomical view of the biliary tree and its relationship to the choledochal cyst [12]. However, as MRI is expensive and takes a longer time to execute, it is not used as frequently as other modalities. So, a high index of suspicion should be present in cases like ours to avoid delays in diagnosis and the devastating complications that accompany such delays.

Choledochal cysts can cause major complications for both the mother and the fetus, which is one of the main challenges that physicians face while managing such cases [8]. Depending on the patient’s status, conservative management options such as US-guided percutaneous drainage should be considered as studies have shown satisfactory results in stabilizing the patient until delivery [13]. However, due to the increased risk of carcinoma, surgical excision of the cyst should be performed only when necessary [14]. Surgical treatment options include decompression with Roux-nY, which showed improvement in 60%-70% of patients; another surgical option is excision with hepaticojejunostomy, which is considered the treatment of choice nowadays for stable non-pregnant patients [15]. However, in type IVA like our patient, the presence of intrahepatic cysts accounts for a number of long-term complications such as hepatolithiasis with recurrent cholangitis, secondary biliary cirrhosis with portal hypertension, and malignant transformation [15]. Excision of the extrahepatic cyst with drainage of the intrahepatic cyst part with wide hilar or subhilar anastomosis has shown satisfactory results in type IVA patients [15].

## Conclusions

The diagnosis of choledochal cyst in adults is a very challenging matter, especially in pregnant patients, due to the hormonal changes of pregnancy, the effects of the gravid uterus, and the limitations of the imaging studies. As a result, physicians should be willing to perform costly investigations that would provide a definitive diagnosis whenever it feels necessary and should prioritize the patient's best interests.
